# Addressing Barriers to Health Care Access of Congenital Heart Disease Patients in Guyana

**DOI:** 10.1177/2333794X211012977

**Published:** 2021-04-29

**Authors:** Sarah Ames, Emma Pillsworth, Arnelle Sparman-Shelto, Debra Lynne Isaac

**Affiliations:** 1 University of Saskatchewan, Saskatchewan, SK, Canada; 2 Northern Ontario School of Medicine, Sudbury, Ontario, Canada; 3 Georgetown Public Hospital Corporation, Georgetown, Guyana; 4 University of Calgary, Calgary, Alberta, Canada

**Keywords:** pediatric cardiac disease, congenital heart disease, global health, barriers to care

## Abstract

In order to mitigate the late presentation and resulting poor outcomes of children with advanced cardiac disease, the Ministry of Public Health (MOPH) in Guyana has expressed interest in identifying ways to improve access to health care for these children. The goal of this study was to identify barriers faced by CHD patients and their families in accessing pediatric cardiology services in Guyana, and to identify limitations to the diagnosis and referral of CHD patients by health care professionals. Two surveys were used to gain insight into the experiences of practicing health care professionals and the parent(s) or guardian(s) of children with CHD. Patients were identified based on convenience sampling at cardiology clinics and outreach clinics in both urban and rural Guyana. Physicians were identified using convenience sampling at health posts in rural Guyana. Fifty-two (n = 52) families were identified and interviewed throughout the regions visited. The majority of families identified distance, the need to travel, and their inability, financially and practically, to attend clinic as the main barrier to accessing specialized care. Twelve (n = 12) health care providers were interviewed. They identified limited knowledge surrounding the diagnosis and management of CHD, and perceived impracticality of referring patients to specialized services, despite being aware of the referral process. This study identifies the need for improved outreach and support for health care providers and families, especially those living in rural communities. It identifies some of the challenges faced in managing patients with CHD in Guyana, while establishing specific areas for quality improvement.

## Highlights

**What do we already know about this topic?**
Identification and management of congenital heart disease poses a significant challenge for countries with limited public health resources.
**How does your research contribute to the field?**
In this paper we identify barriers to congenital heart disease patients and their families to access pediatric cardiology services, discuss obstacles to provision of timely care and reflect on how knowledge of these obstacles can lead to opportunities to overcome them.
**What are your research’s implications toward theory, practice or policy?**
Given the world-wide lack of access to adequate pediatric cardiac care in low income and developing countries, our experiences may guide not only Guyanese policy but potentially apply to the larger global community.

## Background

Congenital heart disease (CHD) or congenital heart defect is defined by the American Heart Association as an abnormality in the way the heart or blood vessels near the heart develop before birth.^1^ CHD accounts for nearly one-third of all major congenital anomalies worldwide. The estimated global birth prevalence is 9.1 per 1000 live births.^2^ This is a significant burden of disease for any country and poses a significant challenge to countries with limited public health resources. Untreated congenital heart disease puts patients at an increased risk for pulmonary hypertension, arrhythmias, infective endocarditis, anticoagulation and congestive heart failure.^3^ These medical conditions compound the burden of disease through increased morbidity and mortality. Although some small cardiac shunts resolve spontaneously with time, more significant CHDs require surgical repair and long-term monitoring throughout childhood and into adulthood. Many patients with CHD also require corrective surgery and/or medical surveillance for comorbid health conditions.

Historically, the Georgetown Public Hospital Corporation (GPHC) had limited access to diagnostic tools and medical expertise required for appropriate identification and management of patients with CHD. Patients accessing specialized services were required to travel outside the country at a high personal cost. Additionally, there was no access to physicians or technicians trained in pediatric cardiology and echocardiography in order to confirm CHD diagnosis and plan for appropriate management, including surgery. This gap in resources and knowledge left a significant number of patients without the necessary medical expertise required to manage children born with CHD. Since 2015, the GPHC has substantially advanced its capabilities in CHD care through the *Guyana Program to Advance Cardiac Care* (GPACC). Through this project the cardiology services available to both pediatric and adults with heart disease has drastically improved, and now includes trained echosonographers, specialized cardiology clinics and connections with international cardiac surgical services. Despite these improvements, patients from rural communities continue to present with missed diagnoses and advanced cardiac disease. Previous studies through GPACC have identified through the comparison of birth prevalence and number of patients with CHD, that there are almost certainly a significant number of children with CHD not being seen by cardiac specialists.^4^ This study additionally supports the concern that rural areas are disproportionally missed in identifying and managing CHD,^4^ despite improvements in cardiac care.

The social and geographic environment in rural Guyana creates additional barriers to accessing advanced medical care for patients with CHD. Less than 10% of the population lives outside the main center of Georgetown and coastal region, spread out in greater than 90% of the geographical area.^5^ This creates a significant challenge for resource allocation and distribution to rural communities. Transportation to many regions in Guyana is limited to boat or air transportation, compounding the cost of resource distribution and transport of families to GPHC. Even those people living in the relatively near to Georgetown coastal regions face issues with lack of affordable public transportation options. The small government-provided health posts, available in rural centers, are often the only available health care option for the individuals living rurally. In larger health posts, inexperienced physicians in their first years since graduation are often stationed. In smaller health posts, medical extension officers (Medex) or midwifes are the senior health care providers for the community.

All of these factors considered, there is a significant need to better understand the barriers experienced by families of patients with CHD, as well as the barriers to health care professionals working with these population. Additionally, there is a need to better understand which barriers are of most significance in order to triage resources and facilitate change in a way that will benefit the greatest number of patients.

## Study Objectives

(1) To identify barriers to CHD patients and their families accessing to pediatric cardiology services in Georgetown.
(2) To identify limitations to health care professional’s appropriate diagnosis and referral of CHD patients.

## Methods

Two surveys were created using both sliding scales and free-text short answers. Questions were created based on consultation with experts on possible barriers to knowledge translation to physicians in rural Guyana, and access to health care for families of patients with CHD. The surveys were then adapted to match appropriate language for families in rural Guyana, with the understanding that a variety of education levels would be present in the population. In addition to standardized questions, each survey allowed for open feedback on the interviewee’s experience, challenges, and suggestions in order to capture themes not represented in the survey.

Families were chosen based on convenience sampling and consented to be interviewed both at the Georgetown Public Hospital Centre pediatric cardiology clinic and rural health posts. Locations were chosen based on access while traveling for outreach clinics. Not all regions were represented. Surveys were administered to families and health care providers by members of the research team in a private location where responses could not be overheard by others

All surveys completed were included as families were only excluded if they did not wish to participate. Consent forms were obtained from each family. Where appropriate, thumb prints were used as replacement for signature when the interviewee was unable to write.

The data was anonymized and then compiled using thematic analysis. Standardized questions were compiled under appropriate themes and descriptive analysis was performed to characterize additional comments prior to being placed under theme headings.

### Ethical Approval and Informed Consent:

Ethics approval for this study was obtained from the University of Calgary Research Ethics Board (REB17-0991) and the Guyana Ministry of Public Health Institutional Review Board (#242). Patients were not exposed to any significant risk by participating in this study. All data collected from the surveys was anonymized and stored in a password protected file. Consent forms were kept in a separate secure location in order to maintain anonymity.

## Results

### Patient Survey Results

Patient demographic information is summarized in Table 1. Fifty-two (n = 52) families were interviewed. Of these 53.8% (n = 28) were from regions 3 and 4, representing families living in relatively close proximity to the urban center of Georgetown. The additional 46.2% (n = 24) were from regions 1, 2, 5, 6, 7, 9, and 10 representing the rural population of Guyana. No surveys were collected from region 8. One survey was performed where the family’s region was not identified. The mean age at diagnosis was 29 months or 2.4 years old. The mean age in the urban area (regions 3 and 4) was 20 months or 1.7 years old, in contrast to the mean age in the rural areas (regions 1, 2, 5, 6, 7, 9, 10, and unknown) which was 58 months or 4.9 years old.

**Table 1.** Demographics of Patients’ Families Interviewed.

Home region	Total number (n = 52)	Percentage	Age at dx (years)	Age at dx (months)^a^
1	2	3.8	7.5	90
2	8	15.4	6.6	80
3	9	17.3	1.0	12
4	19	36.5	2.0	24
5	3	5.8	1.2	14
6	4	7.7	3.0	36
7	3	5.8	9	108
9	1	1.9	1.2	14
10	2	3.8	0.5	6
Unknown	1	3	4.0	48
Mean	52	44.2	2.4	29
Mean urban^b^	28	53.8	1.7	20
Mean rural^c^	24	46.2	4.9	58

a Rounded to the nearest month.

b Urban includes patients from regions 3 and 4.

c Rural includes patients from regions 1, 2, 5, 6, 7, 9, 10s and unknown.

**Figure 1.**
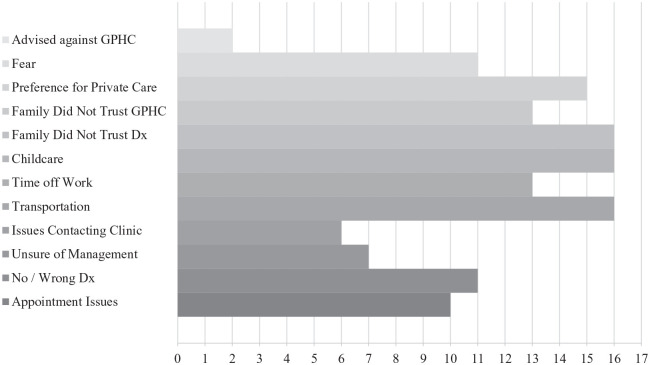
Reasons for delayed presentation to hospital.

Although specific challenges varied depending on the families’ home region and their experience (Figure 1), many of the families expressed avoidance of health care services out of fear of their child’s condition (n = 11) and a misunderstanding regarding the severity of their child’s diagnosis, prognosis, and management plan (n = 16). Some families expressed preference for private hospital care (n = 15) and distrust of the services at the public hospital GPHC (n = 13). Two families (n = 2) reported being advised against attending GPHC by family, friends, or health care professionals. Many families expressed challenges with the practicality of attending clinic due to problems arranging childcare for their other children (n = 16) and getting time off work (n = 13). As the appointments given to families were for a particular day, and not a specific time, families also expressed frustration in scheduling appointments (n = 10) and arranging an entire day off to attend a clinic. On a few occasions, patients described being given a new appointment day, after spending the entire day waiting at the clinic to see a physician. Additionally, parents reported having difficulty contacting the clinic (n = 6) and were required to visit the clinic in person to be given their appointment day. This system required families to present to hospital days in advance to their appointment, leading to additional time away from work, transportation costs, and child-care costs. For rural patients, this was exponentially challenging as families needed to travel well in advance and then remain in Georgetown while awaiting their appointment. In the rural communities, many families expressed great discomfort with the accommodations in Georgetown, limited access to transportation to visit to the hospital (n = 16), and long wait times to return home. The alternative of paying for their transportation and accommodation out of personal funds was not financially possible for many families. Finally, some families reported that they were initially given the wrong diagnosis (n = 11), and 6 families (n = 6) reported being given the correct diagnosis, but their health care team was unsure of the management of their child’s illness. Both scenarios delayed the patients’ presentation to specialized care.

In addition to the structured response questions, families had the opportunity for open feedback on barriers to accessing care at GPHC. The trends from these results are as follows. Of the families who expressed difficulty with arranging child-care, some expressed an inability to pay for a third party to look after their family while attending clinic with the patient. The time off work required to bring a child to the hospital was also a significant barrier as families fear employers will not accepting or supportive of time off for medical reasons of the family and that their jobs were at risk or lost due to time missed. Medication access was also a barrier for families, as medications were not always available in a timely manner or were not provided through the public system.

### Physician Survey Results

Physician demographic information is summarized in Table 2. Twelve (n = 12) first line health care providers were interviewed: 5 community health care workers, 4 medical doctors, 2 midwifes, and 1 Medex. Two (16%) work in urban areas (regions 3 and 4), while the other 10 (84%) work in rural areas (regions 2, 5, 6, 7, and 10). The mean years working after completing training was 6.6 years among all HCPs. The years of experience were swayed by community health care workers with average years worked of 9 years, while the medical doctors had the least experience with mean years worked of 3.5 years.

**Table 2.** Demographics of Health Care Providers (HCP).

Demographic characteristics	Total (n = 12)	Percentage	Years working (mean)
Training			
CHW*	5	42	9
Medical doctor	4	33	3.5
Midwife	2	17	5.5
Medex	1	8	9
Home region			
2	2	16	
3	1	8	
4	1	8	
5	1	8	
6	1	8	
7	5	42	
10	1	8	
Average years working			
Mean			6.6
Median			5

* Community healthcare worker.

**Figure 2.**
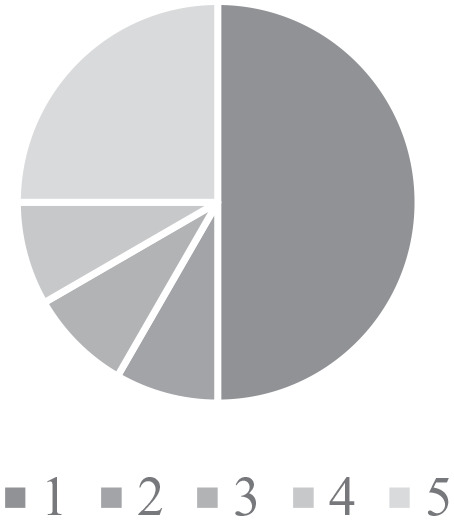
Confidence in identifying CHD. Figures used a scale from 1 (poor) – 5 (excellent) ability in the area of the question.

**Figure 3.**
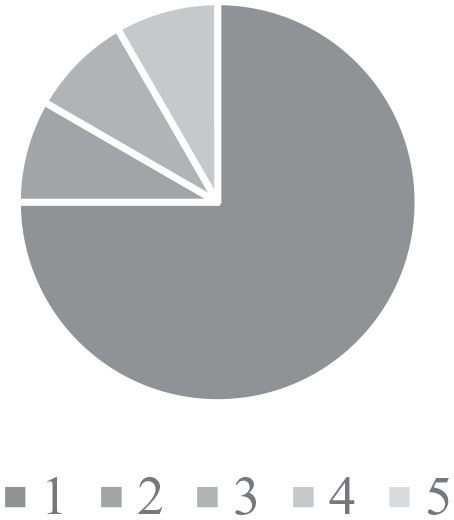
Confidence in managing CHD. Figures used a scale from 1 (poor) – 5 (excellent) ability in the area of the question.

**Figure 4.**
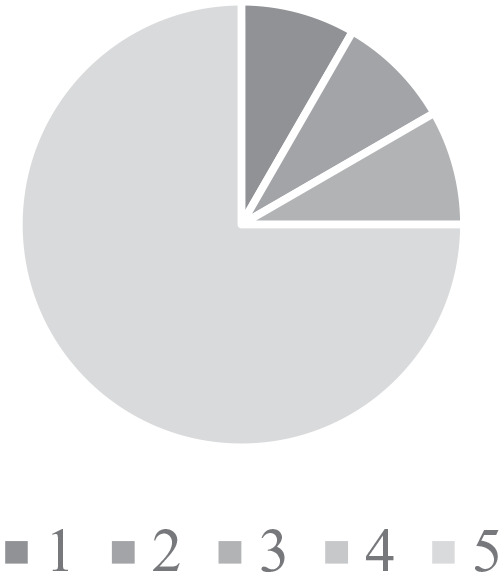
Knowledge of the referral process. Figures used a scale from 1 (poor) – 5 (excellent) ability in the area of the question.

**Figure 5.**
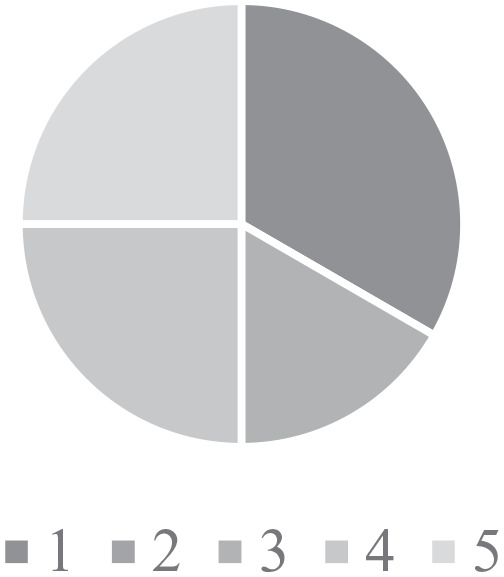
Knowledge of services available. Figures used a scale from 1 (poor) – 5 (excellent) ability in the area of the question.

Findings from the surveys found that 50% of HCPs felt that they had poor (1) confidence in their ability to identifying CHD (Figure 2), and 75% had poor (1) confidence in their ability to in managing CHD (Figure 3). In contrast, HCPs had a good understanding of the referral process to GPHC with 75% of responses rating excellent (5) in response to this question (Figure 4). The knowledge of services available at GPHC was variable with 25% of responses stating that had excellent (5) knowledge, whilst 33% reported poor (1) knowledge (Figure 5).

**Figure 6.**
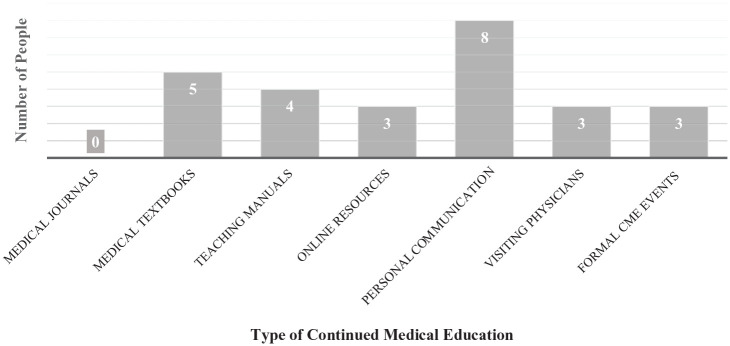
Forms of continued medical education (CME) identified by health care professionals.

Only 3 (n = 3) health care providers (HCPs) felt that they had formal mentorship or training in CHD. Eight (n = 8) used personal communication with experienced physician(s) at GPHC to help advance their knowledge and 3 (n = 3) felt that visiting physicians was a significant source of their current knowledge. Only 3 (n = 3) felt they had access to online resources, and none felt that they had access to current medical journals. Five (n = 5) HCPs used personal medical textbooks from the time of their training to refer to; this primarily being medical doctors. Four (n = 4) HCPs, primarily CHWs, used teaching manuals (n = 4) from the time of their training. These limited resources and rare opportunity for continue medical education (CME) were all identified as possible barriers to advancing the knowledge of HCPs. The most used resources included medical textbooks (n = 5) from their training and personal communication (n = 8) with visiting physicians (Figure 6).

**Figure 7.**
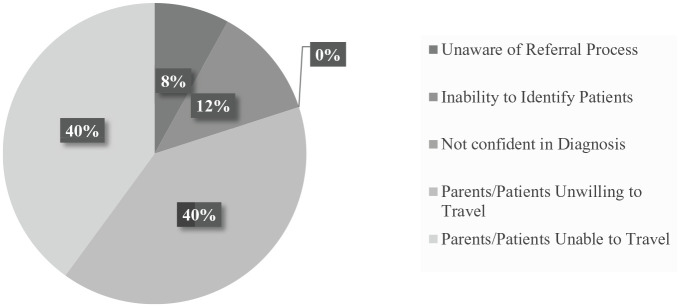
Barriers to referral of patients to GPHC.

Finally, health care providers (HCPs) identified barriers to families accessing specialized services at GPHC (Figure 7). The primary concerns of HCPs were similar to the concerns expressed by families and included apprehension (n = 10), and often refusal (n = 10), to travel to GPHC due to practicality, cost, and concerns about the needs of family members left behind while away. Other barriers included HCPs not being able to identify patients (n = 3) or limited understanding of the referral process (n = 2). All of these concerns additionally extended the length of time to present to specialized care as patients were delayed or missed. No HCPs reported low confidence in their ability to diagnosis CHD.

## Discussion

### Objective 1: Identify Barriers to CHD Patients and Their Families Presenting to Pediatric Cardiology Services in Georgetown

The barriers identified by the survey results from families can be organized into 3 main themes: financial, logistical, and illness perception.

### Financial

Although the hospital and physician services are covered through the public health care system, additional costs are still significant barriers for families. The cost associated with transportation and time off work were identified as barriers to timely access to specialized cardiac care.

In rural communities the financial burden was even more restrictive. Families in these communities reported having less flexible income than those in urban centers, while facing increased financial cost to access medical care. Transportation to rural communities is often limited to ATV and boat, or plane transport. These options for families are either financial extreme (plane travel), or extremely time consuming (ATV/boat). Both options cause significant stress on families and often lead to postponing contact with GPHC until advanced illness was identified. Additionally, families are often required to choose who in the family should take priority in accessing specialized services, as the cost associated cannot be provided for multiple family members. The additional costs felt by rural patients likely plays a role in their presentation to hospital much later in the course of the illness progression compared (average age of 4.9 years old) to urban populations (average age of 1.7 years old).

In order to address this barrier, significant resources and infrastructure are required. This is unlikely to be an easy fix and requires significant planning and a large allocation of funds in an already strained public system. However, increasing the availability of travel stipends, including funds for non-emergent referrals, could significantly reduce the cost, morbidity and mortality of treating patients with late-stage disease and improve continuity of care. Finally, improved logistics around clinic appointments could reduce the amount of time urban and rural patients need to spend at GPHC and potentially lead to reduced overall costs.

### Logistical

In addition to the financial barriers to accessing health care, families expressed concern around the logistics of attending clinic. For urban families, the most common complaint was associated with accessing transportation as the bus system was not available in all areas and many families did not have direct access to a vehicle. This meant that arranging transportation through family and friends was required and understandably more complicated. Childcare was also a significant concern as most families had more than 1 child and was required to arrange supervision for them or bring them along to clinic. While bringing additional family members to clinic may appear appropriate it compounds the cost and practicality of transportation.

In rural communities, logistical issues regarding childcare and transportation were also present. In addition to these barriers, there was the added component of requiring accommodation while in Georgetown. Families expressed great dislike of their experience at these accommodations, so much so that some families refused to return. The housing apartments provided for out of town families are subjectively very uncomfortable and over-crowded. Food services at the apartments were unappealing, and long wait times to return home compounded the resistance to present to hospital. Returning home after attending GPHC was a major logistical barrier expressed by rural families. Planes were scheduled to return only once enough patients had collected to fill a trip, in order to reduce the cost of transporting families back and forth. As many of the communities have a small population, patients expressed frustrating in having to wait weeks to return home.

Logistics surrounding getting an appointment were also limiting for families. The need to attend clinic in person to get a future appointment day double the time and cost for families. Additionally, being given an appointment day rather than a specific time eliminates the option for families to take half a day rather than an entire day off work and arrange for childcare. Finally, the complexity in gaining appointments drastically increases the patients lost to follow up especially those who were rescheduled or cancelled on their appointment day.

Improving appointment scheduling could reduce the families’ burden on time away from work, as well as childcare needs. Families should be able to book appointments over the phone, rather than be required to attend in person, and should be given appointment times in addition to date in order to better plan their attendance. Improved organization of clinics by timed appointments would additionally reduce the incidence of patients presenting to clinic and not being seen, requiring them to attend an additional clinic on another day.

### Illness Perception

Health literacy was an additional barrier identified from survey results. Many patients expressed fear or uncertainty around their child’s diagnosis and prognosis. Some families were not appropriately counseled on their child’s illness and did not understand the severity of the illness or need for medical attention. In contrast, some families understood the consequences and prognosis but felt that nothing could be done to support their child, and therefore did not wish to access medical supports.

The parallel private system also compounds the family’s apprehension in seeking public medical services. Although there is no evidence that the private system has better access to needed medical and surgical expertise, some families maintain the misconception that the private system is the best service for their child. This has led to delays in some families accessing the public system, as they first sought out private clinics before learning of the specialized public care available. Other families delayed their presentation to the public sector out of an inability to afford private care, and a distrust for the public system.

In the rural communities, health literacy was also limited. As many families in these communities rarely if ever interact with specialized medical care, further apprehension around the necessity of these services was expressed. In addition, many of these families felt marginalized and unsupported in the urban communities and in turn feared traveling into Georgetown.

Improving health care providers communication strategies around patient education could begin to address the misconceptions families have around their child’s diagnosis. Additionally, as GPHC is able to provide positive interactions with patients and improve outcomes overtime, families will begin to trust the public system. This would help reduce the delay between patient identification and connection with health services. Additionally, continued focus on connecting with rural populations through rural outreach programs and support of health care providers in these communities will help with the additional barriers faced by this population. Public service announcements on radio and on television, addressing the issue of heart disease in children and the availability of specialized care at GPHC may also improve knowledge and trust.

Summary of Recommendations:
○ Increase travel stipends for rural patients
○ Improve accommodation for rural families staying in Georgetown
○ Allow for appointment scheduling over the phone
○ Arrange clinic days so that families are given appointment times in addition to date
○ Improve health care provider access to education on communication and patient teaching strategies
○ Outreach clinics in major rural centers with adequate advertisement in order to improve connection with specialized care
○ Public service announcements regarding heart disease in children and public services available
○ Cultural competency teaching for physicians in rural and urban settings around indigenous groups in Guyana

### Objective 2: Identify Limitations to Health Care Professional’s Diagnosis and Referral of CHD Patients

Unexpectedly, most of the health care providers (HCPs) surveyed felt knowledgeable about the referral process to GPHC, although many were unclear as to what services were offered once referred. Comfort in identifying patients with CHD was also higher than expected given the relatively low number of patients being referred, which may in itself represent an unrecognized knowledge gap. Comfort in managing patients with CHD independently was limited, again likely due to knowledge limitations of HCPs and their limited access to consulting specialized services. Additionally, the resources for investigation and pharmaceutical therapy is limited and inadequate in most rural centers. At GPHC physicians have access to trained cardiologists and echocardiography to consult and obtain advice on patient care. In contrast, in the rural settings the HCPs are alone in making decisions without the support of imaging or specialist advice. Despite the knowledge gaps, a significant number of HCPs had knowledge enough to diagnose some forms of CHD and were aware of the referral process, suggesting that logistical and illness perception barriers are likely also playing a significant role in the limited referrals from rural settings.

Health care workers in rural communities expressed great interest in further training, either through access to up to date resources or through continued medical education opportunities. With little support to attend further training in Georgetown, many rural HCPs have not had the opportunity to improve their skills or update their knowledge since their time in training. Additionally, most health posts have no internet access, and therefore the HCPs did not have means to access up to date resources themselves. Additionally, the HCPs have strict weight restrictions for their luggage on the rare occasion they leave the small communities. This limits their ability to bring additional resources back with them to their communities in the form of textbooks or medical journals. HCPs also expressed great appreciation for any exposure they had to specialized services through visiting physicians. They felt that the ability to discuss cases when appropriate with specialist through a phone call would significantly improve their abilities to manage patients rurally, and better triage transportation services to those patients most in need.

Supporting HCPs in accessing up to date information through electronic resources on memory drives, providing continued education courses either in person at GPHC or offline programs, and connecting them with specialist services through phone consultation would likely significantly improve their understanding and ability to manage CHD patients in rural settings. Finally, directing funds toward rural settings to support access to basic imaging modalities (x-rays and ultrasound machines) in larger rural settings or outreach clinics could reduce unnecessary referrals and help physicians triage patients at highest risk.

Summary of Recommendations:
○ CME events and resources for health care on CHD identification and management
○ Provide electronic up to date resources (textbooks, journals, etc.) through internet access and/or memory drives
○ Develop a phone consultation service at GPHC to provide recommendations to HCPs
○ Access to outreach imaging clinics including x-ray and ultrasound to help risk stratify patients and direct referral urgency.

### Study Limitations

Surveys were completed based on convenience sample at regions visited by the research team. There are a few regions with no representation in the study. Additionally, many regions were represented by a few voices that may or may not represent the common view of that area. Results need to be evaluated with these limitations in mind, recognizing that the perspectives capture are only representatives of a small number of voices within a complex system.

There were significant language barriers during many of the surveys without access to translation services. Although the official language in Guyana is English, there is significant different dialect between Canadian English and Guyanese English leading to some challenges with communication. The rural population also often did not speak English at all. Additionally, the education level is highly variable within Guyana, with some families unable to read or write while others are university educated. There is a possibility that some responses to questions were made without complete understanding of the question. The study group worked hard to investigate answers in order to reduce this risk, however the possibility is still present.

The survey questions were designed prior to the study based on suspected barriers. This led to the identification of some unexpected barriers within the general feedback section. Although represented in the results, not every participant was asked their perspective on each of these barriers as they were not identified until part way through the study.

Further research is crucial to better evaluating the barriers identified in this study and their importance across the different regions in Guyana. It is likely that specific regions may experience barriers differently based on their geographical location and current supports at their health posts. Additionally, further research into the current identification of CHD in the perinatal and neonatal periods would be helpful in identifying opportunities for improvement in this specific population. Finally, further study will be required to evaluate any quality improvement changes made.

## Conclusion

This study identifies the need for improved outreach and support for health care providers and families, especially those living in rural communities. It demonstrates the challenges faced in identifying and managing patients with congenital heart disease, while capturing specific areas for quality improvement. A focus on parent/patient education at the point of diagnosis, and addressing logistic and financial barriers for families, could reduce the number of patients presenting late in their illness progression. Support for rural health care providers in contact with specialized services, and provision of continuing medical education resources would improve trust and collaboration, while supporting more effective resource allocation. This study identified numerous opportunities to mitigate inequities and improve the access to health care in Guyana for current and future CHD patients. Further study is required to verify these results in a larger sample and identify specific barriers in each region of Guyana.

## Supplemental Material

sj-pdf-1-gph-10.1177_2333794X211012977 – Supplemental material for Addressing Barriers to Health Care Access of Congenital Heart Disease Patients in Guyana Click here for additional data file. Supplemental material, sj-pdf-1-gph-10.1177_2333794X211012977 for Addressing Barriers to Health Care Access of Congenital Heart Disease Patients in Guyana by Sarah Ames, Emma Pillsworth, Arnelle Sparman-Shelto and Debra Lynne Isaac in Global Pediatric Health

sj-pdf-2-gph-10.1177_2333794X211012977 – Supplemental material for Addressing Barriers to Health Care Access of Congenital Heart Disease Patients in Guyana Click here for additional data file. Supplemental material, sj-pdf-2-gph-10.1177_2333794X211012977 for Addressing Barriers to Health Care Access of Congenital Heart Disease Patients in Guyana by Sarah Ames, Emma Pillsworth, Arnelle Sparman-Shelto and Debra Lynne Isaac in Global Pediatric Health
