# Supplementary material for: Addressing Barriers to Health Care Access of Congenital Heart Disease Patients in Guyana
Source: Glob Pediatr Health. 2021 Apr 29;8:2333794X211012977. doi: 10.1177/2333794X211012977 (PMC8107924; doi:10.1177/2333794X211012977)
Supplement: sj-pdf-1-gph-10.1177_2333794X211012977 – Supplemental material for Addressing Barriers to Health Care Access of Congenital Heart Disease Patients in Guyana [file sj-pdf-1-gph-10.1177_2333794X211012977.pdf]

Georgetown Public Hospital Corporation
&
University of Calgary
Physician Questionnaire

In which village / town do you work in Guyana? ____
Region ________________

What is your training? physician ___ nurse ____ medex ___ community health worker ___
How many years have you been practicing medicine since graduation? ___________________________
Where have you practiced? ____
Where did you complete your training? ____

Have you undertaken formal post-medical school residency training? ____________________________
If so, what program and where ____
Do you participate in continued medical education, if so where and how often?___________________
____

On the scale from: 1 (not confident) – 5 (completely confident), how confident are you in your:

1. Your ability to identify pediatric patients with suspected/possible cardiac disease
   1 2 3 4 5
2. Your ability to manage pediatric patients with suspected or confirmed cardiac disease
   1 2 3 4 5
3. Your knowledge of referral process to GHPC pediatric cardiology clinic
   1 2 3 4 5
4. Your knowledge of available services at GHPC for pediatric cardiac disease
   1 2 3 4 5

What form of continued education or reference material do you use to keep up to date with new medicine and support information?
___ training manuals
___ medical journals
___ medical textbooks
___ online resources (which sites) ____
___ personal communication with mentors and specialists
___ visiting physicians
___ formal CME events

What barriers / limitations do you face when attempting to keep up to date or learn new medical material?
____
____

What are the main obstacles faced in referring children with suspected cardiac disease to the Georgetown Pediatric Cardiology Clinic (GPCC)?
__ not aware that there was the option to refer patients to a specialized pediatric cardiac clinic at GPHC
__ not sure who should be referred to this clinic
__ not confident that you can make the diagnosis of heart disease, so reluctant to refer
__ parents / patients unwilling to travel to this clinic
__ parents / patients unable to travel to this clinic
__ other obstacles ____

What support and/or knowledge do you feel you would need (or would be helpful to you) in order to better identify, manage and / or refer pediatric patients with suspected cardiac disease?
