# Supplementary material for: Addressing Barriers to Health Care Access of Congenital Heart Disease Patients in Guyana
Source: Glob Pediatr Health. 2021 Apr 29;8:2333794X211012977. doi: 10.1177/2333794X211012977 (PMC8107924; doi:10.1177/2333794X211012977)
Supplement: sj-pdf-2-gph-10.1177_2333794X211012977 – Supplemental material for Addressing Barriers to Health Care Access of Congenital Heart Disease Patients in Guyana [file sj-pdf-2-gph-10.1177_2333794X211012977.pdf]

# Georgetown Public Hospital Corporation
# &
# University of Calgary
## Pediatric Parent/Guardian Questionnaire

**1.** What is your hometown / village in Guyana? ______________
Region _________________

**2.** When did you first find out that your child had a heart problem? Month __________Year_____

**3.** How old was your child at that time? __________________________________________________

**4.** Who first told you that your child had (or might have) a heart problem:

__ local doctor (name of health centre ___________________________________________________)
__ local nurse (name of health centre ____________________________________________________)
__ local Medex (name of health centre ___________________________________________________)
__ specialist doctor (name of health centre or clinic) ________________________________________)
__ other health care worker ____________________________________________________________)

**5.** When were you told that your child should be sent to Georgetown Public Hospital to be seen in the heart clinic? ____________________________________________________________________

**6.** When was your child first seen at the heart clinic at Georgetown Public Hospital? ______________

**7.** Did you have any difficulties or delays in getting your child seen at the Georgetown Public Hospital heart clinic? _______________________________________________________________________

Please mark any of these that you experienced, and give details if possible:

__ difficult to get your child to be seen by a health care worker (doctor, nurse, medex etc) before you knew that there was a heart problem. Why was this?_________________________________________

__ health care worker did not think your child had a heart problem at first. When was this?
What happened next? _________________________________________________________________

__ Health care worker was not sure how to get more testing and treatment for your child. How did you finally get sent to heart clinic? ______________________________________________________________

__ Difficulty with contact between the local health care worker or the Georgetown Public Hospital and you (for example no phone service). Details __________________________________________________

__ Difficulty getting transportation to go to Georgetown. Please give details (cost, no transportation available, very long distance etc) _________________________________________________________

__ Difficulty getting time away from work to take child to heart clinic.

__ Difficulty finding childcare for other children while you take child to heart clinic.

__ You or your family members did not think your child had a heart problem.

__ You or your family members did not think that there was a good heart clinic at Georgetown Public Hospital.

__ You or your family members thought that your child would get better care in a private hospital or clinic rather than Georgetown Public Hospital.

__ You or your family was afraid of what might happen to your child if a heart problem was found, so you did not want to get the testing done.

__ Someone advised you not to take your child to Georgetown Public Hospital heart clinic. Details: ______________________________________________________________________________________

__ Other things that caused delay or were problems that you had in getting your child seen in the Georgetown Public Hospital heart clinic. Details: ______________________________________________

---

**8.** What do you think could be done to help make sure that all children with heart problems get sent for testing and treatment in a timely manner? __not sure. Possibly have the echo done on the same day the clinic requested it so do not have to bring child back.
